# Digital microscopy and artificial intelligence could profoundly contribute to malaria diagnosis in elimination settings

**DOI:** 10.3389/frai.2022.510483

**Published:** 2022-11-17

**Authors:** Hans-Peter Beck

**Affiliations:** ^1^Department of Medical Parasitology and Infection Biology, Swiss Tropical and Public Health Institute, Allschwil, Switzerland; ^2^University of Basel, Basel, Switzerland

**Keywords:** algorithm, malaria, microscopy, artificial, intelligence

Malaria, transmitted by female Anopheles mosquitoes, is caused by the apicomplexan parasite of the genus *Plasmodium* of which five infect humans, namely *Plasmodium falciparum, P. vivax, P. ovale, P, malariae*, and *P. knowlesi*. It is a life threatening disease of tropical countries around the globe, and more than 600,000 deaths occurred in 2020 (WHO, 2021). More than 80 percent of deaths occur in young children in sub-Saharan Africa mostly caused by the life threatening *P. falciparum*. In general, most deaths from malaria occur in Sub-Saharan Africa (96%) whilst the South-East Asia region accounts for 2.2% of fatal cases and the Eastern European countries for 2.5%. There is also malaria in the Western Pacific region and in the Americas with Brazil having the highest incidence rate (WHO, 2021).

Action for malaria elimination is increasing and in 2021 already 40 previously malaria-endemic countries reported no malaria cases^1^, which is a clear indicator of a move forward to malaria elimination. This success has been possible due to a number of factors but mainly due to distribution of bed nets, availability of effective drugs [artemisinin combination therapy (ACT)] and an improvement of diagnosis by the introduction of rapid diagnostic tests (RDTs).

WHO defines three pillars on which elimination is based on: vector control, rapid and accurate diagnosis, and the availability of rapidly acting effective drugs. There are numerous strategies and possibilities to control the mosquito population with various degrees of success. There are highly effective drugs available [albeit under threat of the development of reduced susceptibility to the currently used artemisinine-combination therapy (Dondorp et al., 2011; Ouji et al., 2018)] contributing to the significant reduction of malaria in endemic areas over the last decade. These drugs are still effective in Africa (Conrad and Rosenthal, 2019) but partial resistance is occurring (Ward et al., 2022) and might have contributed to the recent increase in deaths in 2020 (WHO, 2021).

For diagnosis WHO recommends “prompt parasite-based diagnosis by microscopy or malaria rapid diagnostic test (RDT) in all patients suspected of malaria before antimalarial treatment is administered. Light microscopy entails visualization of the malaria parasites in a thick or thin smear of the patient's blood” (WHO, 2017). ^1^ Microscopy has been used to diagnose malaria since the discovery of the parasites by Alphonse Laveran (Laveran, 1881). Giemsa-stained thick and thin blood films are examined to identify parasites, enumerate them, and to determine the infecting species necessary to guide treatment. This requires very basic laboratory facilities, the availability of quality Giemsa stain, microscopes of sufficient quality, and substantial human skills to identify parasites in the complex mixture of a human blood smear. This procedure is a lengthy and error-prone process and requires substantial human input. Importantly, analysis of Giemsa-stained blood films to identify *Plasmodium* parasites requires extensive experience and constant training and education (expert microscopy).

As these conditions often have been suboptimal, so-called rapid diagnostic tests (RDTs) have been developed around 1995, successfully tested, and finally implemented worldwide (Uguen et al., 1995; Banchongaksorn et al., 1997; Mills et al., 1999). WHO estimates that annually more than 270 million RDTs have been used (WHO, 2017). RDTs detect circulating parasite antigen, in most cases histidine-rich protein 2 and/or 3 (hrp2 and 3) (Uguen et al., 1995) and in some cases parasite-derived lactate dehydrogenase (pLDH) (Piper et al., 1999). A specific aldolase has been identified for *P. vivax* detection as circulating antigen and is being used in discriminating RDTs (Dzakah et al., 2013). RDTs work with capture antibodies that are immobilized as a line on nitrocellulose strips over which blood samples are run by capillary forces. The antigen is then visualized on the line by a secondary antibody initiating a color reaction. Presence of both control and parasite bands indicate the presence of parasites in patient blood. The implementation of RDTs in endemic areas has been a tremendous success. In 2017, in sub-Saharan Africa, RDTs were the most used method to test for malaria among suspected patients in public health facilities. It is estimated that 75% of malaria diagnostic tests were done using RDTs. In contrast, in the same year in Brazil 97% of confirmed cases were diagnosed by microscopy, and similarly in India only estimated 20,000,000 suspected cases were screened with RDTs whilst 114,000,000 were screened by microscopy. In the Americas diagnosis is mostly done by microscopy, the same situation occurs in Eastern European countries were malaria is almost entirely diagnosed by microscopy at heath facilities (WHO, 2020).

Although the implementation of RDTs has been extremely successful and RDTs have tremendously improved the diagnosis of malaria, there are some potential disadvantages and caveats related to this technology. First, there is the possibility of false positives that might be due to the persistence of parasite antigen after successful treatment (Maltha et al., 2013). The major caveat, however, is the possibility of a false negative test result. This can be due to two effects. In the first case too much antigen can prevent binding to the capture and detection antibody due to an effect known as prozone effect (Gillet et al., 2011; Luchavez et al., 2011). In this case, the sample would be considered negative despite the presence of a large number of parasites. Though this is a very rare occurrence. The second reason for a false negative result could be that the target protein (antigen) of the parasite is not produced anymore due to a deletion of the corresponding gene. The first report of such deletion came from Peru in 2010 (Gamboa et al., 2010). By now frequent reports are accumulating that demonstrate that samples remain falsely negative because the parasite hrp2 and/or hrp3 gene has been deleted in the infecting parasite (Koita et al., 2012; Kumar et al., 2013; Deme et al., 2014; Bharti et al., 2016; Beshir et al., 2017; Parr et al., 2017). In some areas, these deletions occur in large numbers, e.g., in Orisha State, India as frequently as in 15% of infected patients (Pati et al., 2018) or in Eritrea where in one hospital 92% of a small number of infections were with parasites carrying hrp2/hrp3 deletions, leading to a large number of false negative tests (Berhane et al., 2018). Although, other RDTs exist that detect *Plasmodium* lactose dehydrogenase (LDH) albeit with lower sensitivity, WHO has called an immediate action to address these problems (Statement by the Malaria Policy Advisory Group on the urgent need to address the high prevalence of pfhrp2/3 gene deletions in the Horn of Africa beyond., 2021). Such failures of RDTs to reliably identify a *Plasmodium* infection can jeopardize all efforts to control or even eliminate malaria.

With this in mind, microscopy will remain the mainstay of parasite-based diagnosis in most endemic areas for health centers and hospitals. This is of particular importance also in areas close to elimination. Although microscopy intrinsically has a rather low limit of detection (10–50 parasites/μl), it hardly can be replaced by highly sensitive molecular tools such as PCR or LAMP (often referred to as Gold Standard), because of the technical requirements and costs. Usually, in elimination areas, index cases (with significant parasitemia) are identified by microscopy. Subsequently, family and close-by living individuals are treated without further confirmation (Zhou et al., 2016). Hence, higher sensitivity is rarely required.

Nevertheless, the quality of microscopy-based diagnosis is often inadequate because of low quality of smearing and staining technology, lack of experience of slide reading and interpretation, low quality of microscopes, and in particular lack of constant quality control. It is interesting that in areas of low endemicity malaria diagnosis is more frequently based on microscopy than on RDT (WHO, 2017). This in turn causes a problem with only a few infections occurring each season, microscopists have no routine and lose experience. This situation also adversely can affect malaria control and elimination.

To overcome these limitations new ideas for diagnostic tools have been put forward (World Health Organization, 2022). Many new developments have gone into trials but often included complicated techniques or required advanced laboratory infrastructure. Within this array of new technologies automated microscopy must be seen as a promising development for malaria diagnosis. Murray et al. (2006) already proposed to use digital microscopy to remotely confirm malaria diagnosis from blood smears. This triggered two developments, one being the development of computing algorithms to identify infected cells from Giemsa blood smears already made by health center staff (Frean, 2008, 2009; Purwar et al., 2011; Linder et al., 2014). Secondly, fully automated systems to identify malaria using digital microscopy were tested in the field or on field prepared smears (Kaewkamnerd et al., 2012; Srivastava et al., 2015; Florin et al., 2018; Dhorda et al., 2019; Gordon et al., 2019; Yoon et al., 2019; Molina et al., 2020). Digital fluorescence microscopy was also tested but due to requirements for special chemicals and hardware, this approach has not reached the field in a broader manner (Florin et al., 2018; Gordon et al., 2019; Yoon et al., 2019, 2021; Holmström et al., 2020; Picot et al., 2022). Common to these developments is that all algorithms of devices were experimental tools and prototypes of which none have been yet commercialized or implemented at larger scale. The major caveat for all the developments using artificial intelligence in malaria diagnosis is the lack of standardized and defined training sets of malaria smears making validation a very difficult task.

Digital microscopy and machine-learned algorithms showed high specificity and in most cases comparable sensitivity to routine (expert) microscopy which has a limit of detection of 10–50 parasites/μl (Manescu et al., 2020). Results often were dependent on smear quality and the ability to detect scanty parasitemia. A major obstacle in all yet published studies using digital microscopy and artificial intelligence was the source of material (blood smears or thick films) because of the large variability of blood smearing and Giemsa staining (Torres et al., 2018; Picot et al., 2022; Manescu et al., 2020). Attempts were made to include automatic smearing and staining to standardize this process and to exclude human error (Choi et al., 2021). Thick films, allowing scanning of a large number of blood cells to increase sensitivity, are rarely used due to the complexity of the images and the large number of disruptors within the image (Yang et al., 2020; Abdurahman et al., 2021). Hence, most systems yet used thin films, significantly reducing the sensitivity of the method. Nevertheless, most published studies reached a close-to or similar sensitivity and specificity as expert microscopy (Das et al., 2022; Knapper et al., 2022).

None of the tools yet have been fully tested in the field and to differentiate between all five *Plasmodium* species that infect humans. However, automated digital microscopy holds the promise to enable health care providers in remote areas to provide expert malaria diagnosis. Although, this might not reach the primary health care centers but might well support training for those centers as well as become reference standard in secondary of tertiary health care centers. It could reduce personnel cost, theoretically could be timesaving, and does not require constant training with an in-build quality assurance. An added benefit of digitalization of malaria diagnosis would be the direct linking of results to central databases such as DHIS^2^. Thus, the technology using digital image acquisition and machine learning or artificial intelligence for malaria diagnosis might overcome a number of major limitations observed with RDTs or human routine diagnosis.

For example, RDTs are not quantitative nor are they able to distinguish the transmissible forms, gametocytes, which is important information in our endeavors to curb the disease. Human microscopists become tired and lose experience, automatic devices will not become tired nor do they lose their experience. Finally, such innovative devices must be extremely robust and reliable, must be partially independent of electrical power (battery powered), and most importantly must allow a diagnostic test at affordable costs for developing countries. For the latter requirement, it might be that innovative funding schemes could support such development.

In conclusion, microscopy as diagnostic test for malaria will remain despite other means of diagnosis such as RDTs, because of its ability to enumerate parasitemia and to identify all five species, as well as to detect the transmissible gametocytes. Ideally, to overcome human limitations, automatic microscopic devices with embedded artificial intelligence would provide the same or better quality of results (in particularly with constant improving) and could eventually become the reference for malaria diagnosis based on microscopic examination in endemic areas.

## Author contributions

The author confirms being the sole contributor of this work and has approved it for publication.

## Conflict of interest

Author HP-B acts as scientific advisor to the company Noul Co., Ltd.

## Publisher's note

All claims expressed in this article are solely those of the authors and do not necessarily represent those of their affiliated organizations, or those of the publisher, the editors and the reviewers. Any product that may be evaluated in this article, or claim that may be made by its manufacturer, is not guaranteed or endorsed by the publisher.

## Footnotes

^1^https://www.who.int/teams/global-malaria-programme/elimination/countries-and-territories-certified-malaria-free-by-who (accessed last 03. 01. 2022).

^2^https://dhis2.org/ (accessed last 03.01.2022).
